# Diagnostic feature of tuberculous peritonitis in patients with cirrhosis: A matched case-control study

**DOI:** 10.3892/etm.2014.1538

**Published:** 2014-02-12

**Authors:** HAI-JUN HUANG, JIN YANG, YI-CHENG HUANG, HONG-YING PAN, HONG WANG, ZHUO-CHAO REN

**Affiliations:** 1 Department of Infectious Disease, Zhejiang Provincial People’s Hospital, Hangzhou, Zheijiang 310014, P.R. China; 2 Department of Medicine, Blood Center of Zhejiang Province, Hangzhou, Zheijiang 310061, P.R. China; 3 Transform Medicine Center, Affiliated Hospital of Hangzhou Normal University, Hangzhou, Zheijiang 310015, P.R. China; 4 Department of Respiratory Medicine, Zhejiang Provincial People’s Hospital, Hangzhou, Zheijiang 310014, P.R. China

**Keywords:** tuberculous peritonitis, spontaneous bacterial peritonitis, liver cirrhosis

## Abstract

The aim of the present study was to compare the clinical characteristics of tuberculous peritonitis (TBP) and spontaneous bacterial peritonitis (SBP) in patients with cirrhosis. A retrospective, matched case-control study was conducted consisting of 12 patients with cirrhosis diagnosed with TBP between 2008 and 2011. Control subjects were patients with SBP. Clinical features and laboratory data were analyzed. Compared with SBP, TBP in patients with cirrhosis was frequently associated with extraperitoneal tuberculosis (25 vs. 0%), a more insidious onset (39.67±30.00 vs. 21.60±21.50 days; P<0.05), Child-Pugh classification B at onset (67 vs. 32%; P<0.05) and lymphopenia (0.67±0.22 vs. 1.19±0.41×10^9^/l; P<0.01). Patients with TBP tended to have lymphocytic predominance in the peritoneal fluid (92%), while patients with SBP tended to have neutrophil predominance (68%). Compared with the SBP group, the TBP group had significantly higher ascitic protein, adenosine deaminase (ADA) and lactate dehydrogenase (LDH) levels. Ascitic protein levels were >25 g/l in 9 patients (75%) in the TBP group and in 2 patients (8%) in the SBP group; ascitic ADA activity levels were >27 U/l in 8 patients (67%) in the TBP group, but were not >27 U/l in any of the patients in the SBP group; ascitic LDH levels were >90 U/l in 10 patients (83%) in the TBP group and 5 patients (20%) in the SBP group. Therefore, the results of the present study indicate that TBP should be considered in cirrhotic patients with relevant clinical manifestations and characteristics of laboratory observations.

## Introduction

In previous years, there has been a global increase in the incidence of tuberculosis (TB), along with the prevalence of acquired immunodeficiency syndrome, and the emergence of multidrug-resistant strains. Tuberculous peritonitis (TBP) is primarily caused by hematogenous spread and rarely results from the contagious spread of an infected bowel or fallopian tubes (1,2). It is estimated that TBP represents 4–10% of all extrapulmonary TB cases (3,4).

Diagnosis of TBP is difficult since the clinical features are nonspecific and ascitic fluid may contain few tubercle bacilli that can neither be observed nor cultured. Undiagnosed and untreated TBP results in a mortality rate of 50–60% (5); however, the disease is usually curable when treated properly. Patients with liver cirrhosis are at an increased risk of developing TBP (6,7). TBP in cirrhotic patients can mimic spontaneous bacterial peritonitis (SBP) and is frequently not considered in differential diagnosis, resulting in delayed diagnosis and even mortality (7). Awareness of the clinical features of TBP in patients with cirrhosis is crucial for improving diagnostic accuracy and survival. However, the study of TBP characteristics in patients with cirrhosis, compared with those of SBP, is limited (8). Therefore, a retrospective, matched case-control study was performed to compare the clinical characteristics of TBP and SBP in patients with cirrhosis.

## Materials and methods

### Patient selection

In this retrospective study, the hospital records of 12 patients with cirrhosis who were diagnosed with TBP in the Zhejiang Provincial People’s Hospital (Hangzhou, China) between 2008 and 2011 were reviewed. For the purpose of comparison, 25 patients with definite SBP were selected that matched the TBP patients in age and gender during the same period. The study was approved by the Human Ethics Committee of Zhejiang Provincial People’s Hospital. Written informed consent was obtained from the patient’s family.

### Methods

The diagnosis of liver cirrhosis was confirmed by clinical observations, image analysis or the presence of esophagogastric varices. The severity of liver cirrhosis was graded according to the Child-Pugh classification. Patients with human immunodeficiency virus coinfection or hepatocellular carcinoma were excluded.

All 12 cirrhotic patients with compatible symptoms, including fever, abdominal pain and distention, were diagnosed with TBP if one or more of the following criteria was met: i) Positive culture of *Mycobacterium tuberculosis* from ascites; ii) positive detection of acid-fast bacilli in ascites; iii) demonstration of caseating granulomata in histological examination of peritoneal biopsy specimens; iv) positive detection of *Mycobacterium tuberculosis* in ascites after polymerase chain reaction (PCR); and v) response to antituberculous therapy.

All 25 patients with cirrhosis and clinical manifestations of SBP were diagnosed with definite SBP, defined as SBP caused by one monobacteria (culture was positive for ascites) and a polymorphonuclear leukocyte count in the ascitic fluid of ≥250 cells/μl. Patients with suspected secondary peritonitis were excluded, as discussed by Rimola *et al* (9).

Patient demographics, clinical manifestations, presence of extraperitoneal tuberculosis, hematological data, ascetic fluid analysis and the culture of ascites for bacteria were recorded. The culture of biopsies or ascitic fluid for *Mycobacterium tuberculosis* was not performed.

### Statistical analyses

Proportions were compared using the χ^2^ test or a two-tailed Fisher’s exact test. Continuous variables were compared using the Student’s t-test or the Mann-Whitney U test. Statistical analyses were performed using SPSS software, version 12.0 (SPSS, Inc., Chicago, IL, USA). P<0.05 was considered to indicate a statistically significant difference.

## Results

### Demographic and clinical manifestations

Demographic and clinical characteristics of the 37 participants are shown in Table I. In the TBP group, three cases demonstrated caseating granulomata following histological assessment, two cases showed a positive culture of *Mycobacterium tuberculosis*, two cases detected positive for acid-fast bacilli and in the remaining five patients, diagnosis was based on the positive result of PCR and the response to antituberculous therapy. Of the 25 patients in the SBP group, 12 cases were infected with *Escherichia coli*, seven cases were infected with *Klebsiella* species, three cases were infected with *Streptococcus* species, two cases were infected with *Staphylococcus* species and one case was infected with *Aeromonas* species. The frequency of Child-Pugh class B was significantly higher in the TBP group when compared with the SBP group [8/12 patients (67%) vs. 8/25 patients (32%); P<0.05]. Three cases (25%) in the TBP group exhibited pulmonary TB, but no case was identified in the SBP group. A statistically significant increase in the median duration of symptoms prior to presentation was observed in the TBP group (39.67±30.00 vs. 21.60±21.50 days; P<0.05). There were no other statistically significant differences between the groups with regard to age, gender, etiology of cirrhosis and initial clinical symptoms.

### Laboratory observations

**Figure 1:**
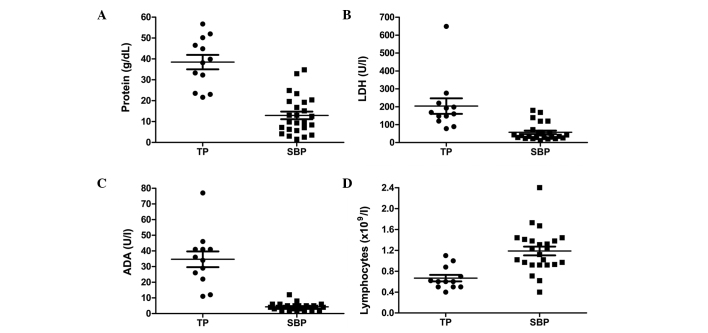
Ascitic and hematological observations of patients with TBP and SBP. The TBP group showed (A) higher protein concentrations, (B) higher LDH activity levels and (C) ADA activity levels in ascites and (D) a lower mean hematological lymphocyte cell count. TBP, tuberculous peritonitis; SBP, spontaneous bacterial peritonitis; LDH, lactate dehydrogenase; ADA, adenosine deaminase.

Laboratory observations are summarized in Table II and Fig. 1. The mean peripheral total white cell count did not differ significantly between the two groups, but the lymphocyte population was significantly decreased in the TBP group when compared with the SBP group (0.67±0.22×10^9^/l vs. 1.19±0.41×10^9^/l; P<0.01). The serum levels of cancer antigen (CA)-125 in the two groups were elevated, but no significant difference was observed. The mean serum protein and albumin concentrations were significantly higher in the TBP group. In addition, the ascitic total white cell count was increased in the TBP group when compared with the SBP group, but no significant difference was observed. However, the proportion of white blood cells was statistically different with 11 cases (92%) with lymphocytic predominance in the TBP group and 17 cases (68%) with neutrophil predominance in the SBP group. Patients in the TBP group had significantly higher ascitic protein, adenosine deaminase (ADA) and lactate dehydrogenase (LDH) levels when compared with those in the SBP group, whereas the distribution of the serum ascites albumin gradient did not differ between the two groups. The ascitic protein level was >25 g/l in 9 patients (75%) in the TBP group (range, 21.62–56.70 g/l) and 2 patients (8%) in the SBP group (range, 1.50–34.80 g/l). Ascitic ADA activity levels were >27 U/l in 8 patients (67%) in the TBP group (range, 11–77 U/l), but no patients in the SBP group had levels >27 U/l (range, 2–12 U/l). The ascitic LDH level was >90 U/l in 10 patients (83%) in the TBP group (range, 78–649 U/l) and 5 patients (20%) in the SBP group (range, 11–180 U/l).

## Discussion

The therapeutic techniques to treat TBP and SBP differ largely. TBP requires conservative quadruple antituberculosis treatment, while SBP requires empirical antimicrobial therapy. However, the diagnostic complications of TBP presents a technical hindrance for effective therapy for these patients.

TBP in patients with cirrhosis presents with nonspecific signs and symptoms, including abdominal distension, fever, abdominal pain and diarrhea, and hence mimics those of SBP. In the present study, it was identified that the clinical symptoms are similar between TBP and SBP in patients with cirrhosis. The onset of TBP is often insidious, even in patients with cirrhosis (7,8). Consistent with a previous study, the median duration of symptoms prior to presentation was >1 month for cirrhotic patients with TBP (8). All patients were examined for signs of TB at additional sites and three cases (25%) in the TBP group were diagnosed with pulmonary TB, but no cases were identified in the SBP group. Therefore, examination for TB at additional sites is important for diagnosing TBP.

Numerous studies have demonstrated that SBP occurs mainly in cirrhotic patients with Child-Pugh class C (10,11), with only one study demonstrating that TBP occurs primarily in cirrhotic patients with Child-Pugh class B (8). The present study revealed similar results with 17 cases (68%) of Child-Pugh class C in the SBP group and 8 cases (67%) of Child-Pugh class B in the TBP group. The present results also indicate that compared with SBP, TBP may develop relatively early in the course of cirrhosis. In addition, the higher protein and albumin concentrations in the serum of cirrhotic patients with TBP may elucidate why the Child-Pugh class is mainly type B in the TBP group.

Notably, there was a significant decrease in the number of lymphocytes in the peripheral blood of cirrhotic patients with TBP. CD4+ T-lymphopenia is considered to be a reaction of mycobacterial infection and not a manifestation of underlying secondary immunodeficiency (12). Chau *et al* (13) hypothesized that sequestration of lymphocytes in the peritoneum may result in lymphopenia in the peripheral blood during a later phase of TBP. Therefore, lymphopenia in the peripheral blood may function as a marker for TBP.

Previous studies have shown that an elevation of serum CA-125 levels may be used as a novel marker for the diagnosis and follow-up of patients with TBP (14,15). In the present study, the serum levels of CA-125 in the two groups were found to be elevated, however, no significant difference was observed. These results were not comparable with earlier observations since there have been no previous studies on the advantages of determining serum CA-125 levels for the diagnosis of TBP and SBP.

The predominance of lymphocytes in ascites is a characteristic of TBP (16). In the present study, 11 cases (92%) were identified to have lymphocytic predominance in the TBP group, while 17 cases (68%) had neutrophil predominance in the SBP group. Therefore, we hypothesized that the aforementioned ascitic fluid features may be a good indicator for diagnosis. In addition, patients in the TBP group were observed to have significantly higher ascitic fluid total protein levels when compared with the SBP group. Several studies have demonstrated that patients with an ascitic protein level of >25 g/l have a high sensitivity for TBP (6,8). In the present study, the ascitic protein level was >25 g/l in 9 patients (75%) in the TBP group, but only in 2 patients (8%) in the SBP group. The protein concentration in the ascites of cirrhotic patients with SBP was ~13 g/l (17). Therefore, a higher protein concentration in the ascites may be considered as a useful marker for the diagnosis of TBP.

ADA has been investigated as a rapid diagnostic tool for TBP (18), however, the role of ADA in the setting of cirrhosis is controversial (19,20). Hillebrand *et al* (19) identified that ADA activity showed imperfect specificity and low sensitivity in cirrhotic patients with TBP. These observations were countered by Liao *et al* (20), who reported that ADA activity showed a high specificity and sensitivity in those patients using a cut-off value of >27 U/l. In the present study, the mean ascitic ADA activity level in 12 patients with cirrhosis and TBP was 35.58 U/l and 8 of these patients had ADA activity level >27 U/l. The maximum ascitic ADA activity level in the 25 patients with SBP was 12 U/l with a mean of 4.32 U/l. Therefore, we hypothesize that the examination of ADA activity is a critical test for diagnosing TBP.

Ascitic LDH levels increased due to the release of LDH from neutrophils (16). Elevation of ascitic LDH may be associated with numerous diseases, including TBP (6,7) and SBP (21). A previous study has demonstrated that an ascitic LDH level of >90 U/l is a useful parameter with high sensitivity and low specificity for the screening of TBP, irrespective of the presence of liver cirrhosis (6). In the present study, the mean ascitic LDH level in the TBP group was 204 U/l and 10 of these patients showed an LDH level of >90 U/l. The maximum ascetic LDH level in the SBP group was 180 U/l with a mean of 57 U/l. Therefore, in a clinical setting, this parameter may be useful in discriminating against TBP.

It is important to be aware of the possibility of TBP in cirrhotic patients with ascites, including patients with known portal hypertension or SBP. In conclusion, clinical features and elevated serum CA-125 levels may not be specific in differentiating from SBP. However, TBP should be considered with the following criteria: Cirrhotic patients with Child Pugh class B; TB identified at additional sites; lymphopenia in the peripheral blood; an ascitic protein concentration of >25 g/l; a predominance of lymphocytes in ascites; ascitic ADA activity levels of >27 U/l; and ascitic LDH levels of >90 U/l.

## Figures and Tables

**Table I:** Demographic and clinical characteristics of the study population.

Characteristics	TBP (n=12)	SBP (n=25)	P-value
Age, years	58.75±12.66^a^	57.84±14.19^a^	NS
Male, n (%)	8 (67)	16 (64)	NS
Etiology of cirrhosis, n (%)			
Hepatitis B virus	8 (67)	17 (68)	NS
Hepatitis C virus	0	1 (4)	NS
Alcohol	3 (25)	3 (12)	NS
Schistosome	1 (8)	2 (8)	NS
Biliary	0	2 (8)	NS
Child-Pugh class, n (%)			<0.05
B	8 (67)	8 (32)	
C	4 (33)	17 (68)	
Tuberculosis at other site, n (%)	3 (25)	0 (0)	<0.01
Duration of symptoms before presentation, days	39.67±30.00^a^	21.60±21.50^a^	<0.05
Initial symptoms, n (%)			
Abdominal distension	11 (92)	23 (92)	NS
Fever	6 (50)	8 (32)	NS
Abdominal pain	5 (42)	9 (36)	NS
Diarrhea	3 (25)	6 (24)	NS

a Values are expressed as the mean ± SD.

NS, not significant; TBP, tuberculous peritonitis; SBP, spontaneous bacterial peritonitis.

**Table II:** Laboratory observations of patients with TBP and SBP.

Parameters	TBP (n=12)	SBP (n=25)	P-value
Hematological observations upon admission			
White cell count, 10^9^/l	4.83±1.45^a^	7.58±5.78^a^	NS
Lymphocyte, 10^9^/l	0.67±0.22^a^	1.19±0.41^a^	<0.01
Protein, g/l	71.19±7.28^a^	63.90±8.92^a^	<0.05
Albumin, g/l	31.62±5.08^a^	27.48±4.16^a^	<0.05
CA-125, U/ml	594±504^a^	439±340^a^	NS
Ascitic fluid observations upon admission			
White cell count, μl	1840±1503^a^	1390±1912^a^	NS
Lymphocyte predominant, n (%)	11 (92)	1 (4)	<0.01
Neutrophil predominant, n (%)	0	17 (68)	<0.01
Monocyte predominant, n (%)	0	4 (16)	NS
Equivocal, n (%)	1 (8)	3 (12)	NS
Protein, g/dl	38.50±11.96^a^	12.94±9.16^a^	0
>25 g/l, n (%)	9 (75)	2 (8)	0
SAAG			
≥11 g/l	10 (83)	22 (88)	NS
ADA, U/l	34.67±17.54^a^	4.32±2.25^a^	0
≥30 U/l, n (%)	8 (67)	0	0
LDH, U/l	203.83±150.55^a^	57.44±48.06^a^	0
≥90 U/l	10 (83)	5 (20)	0

a Values are expressed as the mean ± SD.

NS, not significant; TBP, tuberculous peritonitis; SBP, spontaneous bacterial peritonitis; SAAG, serum ascites albumin gradient; ADA, adenosine deaminase; LDH, lactate dehydrogenase; CA-125, cancer antigen 125.
